# Dupilumab-Induced Scalp Psoriasis in a Patient With Prurigo Nodularis: A Case Report

**DOI:** 10.7759/cureus.37992

**Published:** 2023-04-22

**Authors:** Khalid Al Hawsawi, Amal W AlDoboke, Seham A Alsulami, Ghadeer E Alamri, Raed F Alsufi

**Affiliations:** 1 Dermatology, King Abdulaziz Hospital, Makkah, SAU; 2 Medicine and Surgery, Umm Al-Qura University, Makkah, SAU; 3 College of Medicine, Umm Al-Qura University, Makkah, SAU

**Keywords:** drug-related side effects and adverse reactions, side effect, scalp psoriasis, prurigo nodularis, dupilumab

## Abstract

There are few reports of dupilumab-induced psoriasis recently published. Here, we present a case of a 50-year-old female with a three-month history of persistent itchy scalp lesions. She had an unremarkable past medical history except that she was diagnosed with prurigo nodularis (PN) three years ago and was on dupilumab treatment for one year. Skin examination revealed multiple silvery scaly plaques on her scalp. The examination of the nails and mucous membranes was normal; there were no skin lesions. Based on the above clinical findings, the patient was diagnosed with dupilumab-induced scalp psoriasis. Dupilumab was stopped. Anti-psoriasis treatment (0.05% betamethasone dipropionate-calcepitriol gel) was started and the patient showed improvement. She was put under periodic follow-up.

## Introduction

Dupilumab is a Food and Drug Administration (FDA)-approved injectable biological medication for severe atopic dermatitis (AD), severe bronchial asthma, chronic rhinosinusitis with nasal polyposis, and prurigo nodularis (PN) [1]. It is a human monoclonal antibody of the immunoglobulin G4 subclass that particularly binds to IL-4 and IL-13 receptor complexes to suppress their signalling [2]. PN is an uncommon chronic pruritic skin disorder characterized by numerous, hard, flesh-to-pink-colored nodules along the extensor surface of extremities [3]. We present a case of scalp psoriasis that developed one year after starting dupilumab treatment for PN.

## Case presentation

**Figure 1:**
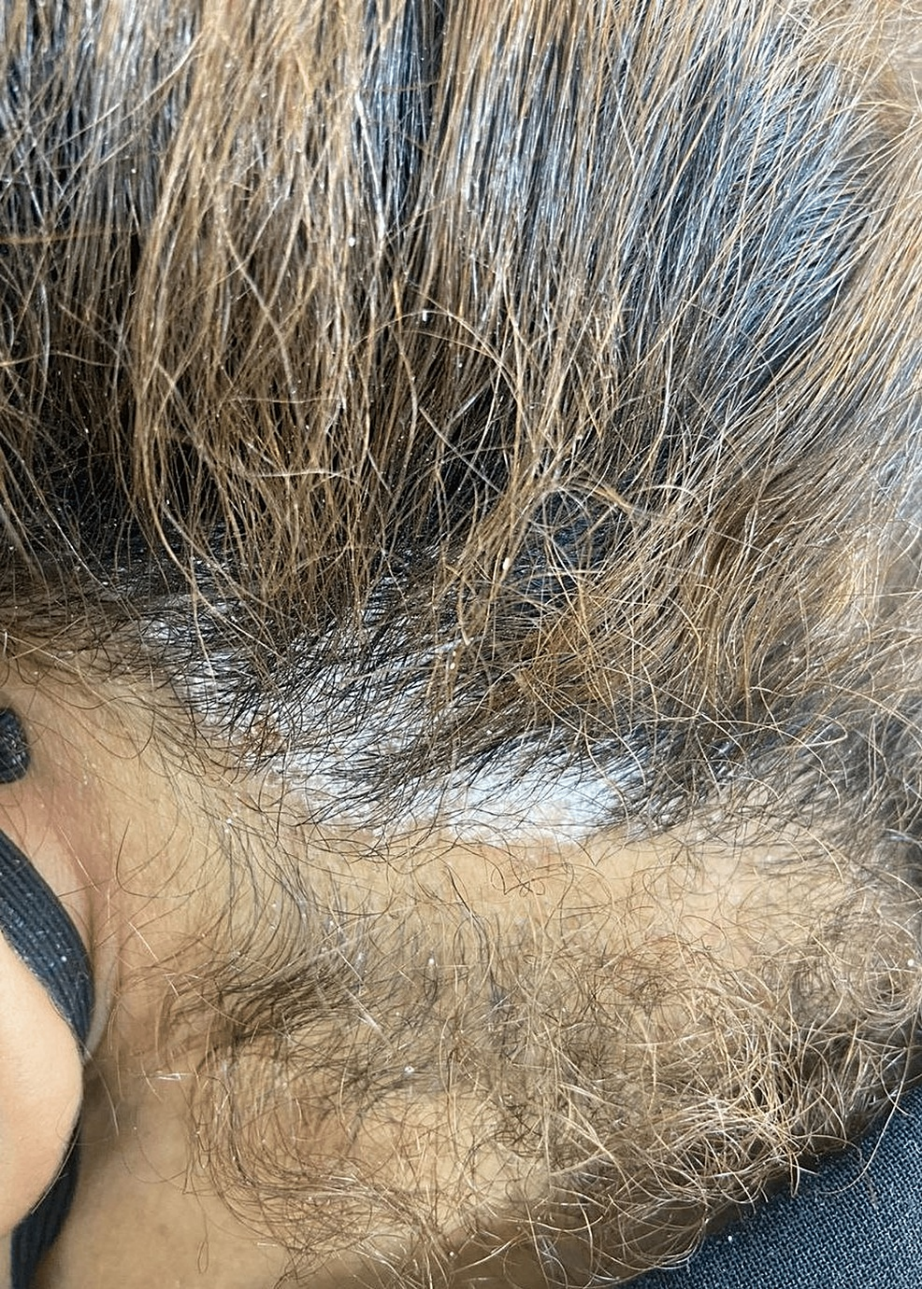
Scalp psoriasis Silvery scaly plaques on the patient's scalp.

A 50-year-old female presented with a three-month history of itchy scalp lesions. She was diagnosed with PN three years ago. She had been using escitalopram 20 mg, betamethasone dipropionate 0.5% ointment, cyclosporine capsules, methotrexate tablets, and narrow-band ultraviolet-B (NB-UVB) phototherapy with variable results. When she presented to us, she was on dupilumab SC injection 300 mg every two weeks for one year with excellent response. She was otherwise in good health with no significant past medical history, drug history, family history, or systemic symptoms. She had no history of joint pain. Skin examination revealed multiple silvery scaly plaques on her scalp (Figure 1). Nail and mucous membrane examinations were normal. No skin lesions were present. Based on the above clinical findings, the patient was diagnosed with dupilumab-induced scalp psoriasis. Dupilumab was stopped. Anti-psoriasis treatment with 0.05% betamethasone dipropionate-calcipotriol gel was started, and the patient showed improvement after two months. She was put under periodic follow-up; she has not experienced any PN flare-ups so far.

## Discussion

Psoriasis is a multifactorial chronic inflammatory autoimmune skin disease with systemic symptoms. The etiology of psoriasis is influenced by the interaction of hereditary and environmental factors [4]. The scalp is one of the most common locations of psoriasis, accounting for 79% of all cases [5]. Although it can develop at any age, it most frequently affects people between the ages of 15 and 30 [4,6]. Unlike skin psoriasis, the treatment of scalp psoriasis is difficult. It includes topical corticosteroids, vitamin D analogues, tazarotene methotrexate, cyclosporine, NB-UVB, and biologic treatment [7]. Although treatment of scalp psoriasis is difficult, our case showed improvement probably because the offending agent was stopped.

As dupilumab is a new biologic that has been approved on September 2022, by the FDA for the treatment of PN, with time, a new side effect might appear [1,3]. Recently, Boudreaux reported five cases of patients with AD who used dupilumab and developed psoriasiform dermatitis during the course of treatment [8]. Tracey et al. and Safa reported cases of erythrodermic and plaque psoriasis in patients who received dupilumab [9,10].

The exact mechanism has not been fully understood until now. AD and PN are mediated through the T helper type 2 (TH-2) pathway, whereas psoriasis is mediated through the TH-1/TH-17 pathways [8]. Dupilumab blocks IL-4 (TH-2 pathway). As a result, dupilumab activates the TH-1/TH-17 pathway, which explains how dupilumab causes psoriasis [10].

## Conclusions

We reported scalp psoriasis as a side effect of dupilumab for treating PN. The exact mechanism is not fully understood. Therefore, further research is required to identify the role of dupilumab in the pathogenesis of psoriasis. This case report aims to increase the perception of dupilumab's uncommon side effects.
